# Development and Comparison of Multimodal Models for Preoperative Prediction of Outcomes After Endovascular Aneurysm Repair

**DOI:** 10.3389/fcvm.2022.870132

**Published:** 2022-04-26

**Authors:** Yonggang Wang, Min Zhou, Yong Ding, Xu Li, Zhenyu Zhou, Zhenyu Shi, Weiguo Fu

**Affiliations:** Department of Vascular Surgery, Zhongshan Hospital, Institute of Vascular Surgery, Fudan University, National Clinical Research Center for Interventional Medicine, Shanghai, China

**Keywords:** abdominal aortic aneurysm, endovascular repair (EVAR), multimodal, morphologic features, deep learning, radiomics

## Abstract

**Objective:**

The aim of this study was to develop and compare multimodal models for predicting outcomes after endovascular abdominal aortic aneurysm repair (EVAR) based on morphological, deep learning (DL), and radiomic features.

**Methods:**

We retrospectively reviewed 979 patients (January 2010—December 2019) with infrarenal abdominal aortic aneurysms (AAAs) who underwent elective EVAR procedures. A total of 486 patients (January 2010–December 2015) were used for morphological feature model development and optimization. Univariable and multivariable analyses were conducted to determine significant morphological features of EVAR-related severe adverse events (SAEs) and to build a morphological feature model based on different machine learning algorithms. Subsequently, to develop the morphological feature model more easily and better compare with other modal models, 340 patients of AAA with intraluminal thrombosis (ILT) were used for automatic segmentation of ILT based on deep convolutional neural networks (DCNNs). Notably, 493 patients (January 2016–December 2019) were used for the development and comparison of multimodal models (optimized morphological feature, DL, and radiomic models). Of note, 80% of patients were classified as the training set and 20% of patients were classified as the test set. The area under the curve (AUC) was used to evaluate the predictive abilities of different modal models.

**Results:**

The mean age of the patients was 69.9 years, the mean follow-up was 54 months, and 307 (31.4%) patients experienced SAEs. Statistical analysis revealed that short neck, angulated neck, conical neck, ILT, ILT percentage ≥51.6%, luminal calcification, double iliac sign, and common iliac artery index ≥1.255 were associated with SAEs. The morphological feature model based on the support vector machine had a better predictive performance with an AUC of 0.76, an accuracy of 0.76, and an F1 score of 0.82. Our DCNN model achieved a mean intersection over union score of more than 90.78% for the segmentation of ILT and AAA aortic lumen. The multimodal model result showed that the radiomic model based on logistics regression had better predictive performance (AUC 0.93, accuracy 0.86, and F1 score 0.91) than the optimized morphological feature model (AUC 0.62, accuracy 0.69, and F1 score 0.81) and the DL model (AUC 0.82, accuracy 0.85, and F1 score 0.89).

**Conclusion:**

The radiomic model has better predictive performance for patient status after EVAR. The morphological feature model and DL model have their own advantages and could also be used to predict outcomes after EVAR.

## Introduction

Endovascular aneurysm repair (EVAR) for abdominal aortic aneurysm (AAA) has some advantages in the minimal invasion, rapid postoperative recovery, shorter hospital stay, and low mortality and morbidity. However, long-term follow-up results reveal a high risk of postoperative complications and re-intervention, when compared with open surgical repair (OSR). Thus, for patients who undergo EVAR, lifetime follow-up is recommended by guidelines (1–4). However, close follow-up may result in unexpected side effects, such as renal function impairment, radiation exposure, and economic and time costs. Therefore, an individualized follow-up protocol is needed (5–9).

Morphological features have been widely used to evaluate risk after EVAR. The operators first treat patients they do not make an estimation of individual patient risk through these adverse morphological features, such as the short neck, angulated neck, intraluminal thrombosis (ILT), calcification, and iliac artery tortuosity (9–11). Recently, Karthikesalingam Alan used preoperative morphological features and artificial neural network (ANN) to predict endograft complications, and Ali Kordzadeh applied 26 preoperative attributes and ANN for the detection of endoleaks (types I–III, respectively). However, the limited number of patients with different complications may have influenced the prediction performance. Moreover, the above studies did not fully exploit the morphological features and did not compare the differences between different machine learning (ML) algorithms, and the feature extraction process was time-consuming (12, 13).

Convolutional neural networks (CNNs) extract non-linear, entangled, and representative features from massive and high-dimensional data from medical images. Benefiting from its strong feature-learning ability, the deep learning (DL) model has shown human expert-level performance in diagnosis, detection, prognosis, and treatment selection (14–16). Unfortunately, preoperative computed tomography angiography (CTA) DL feature stratification methods for EVAR were still unavailable. Radiomics has been widely used in the study of oncology (17). Texture analysis, as a part of radiomics, has gradually been used in AAA, such as predicting expansion, endotension, regression of endoleaks, and aneurysm progression after EVAR (18–21). Compared with texture analysis, radiomics can obtain more features, which may potentially improve prognostic and predictive accuracy in EVAR (22–24). Recently, Charalambous Stavros used radiomics and support vector machines (SVM) trained on 1-month and 6-month radiomic data after EVAR to predict Type 2 endoleaks (T2ELs) leading sac expansion at 1 year (25). Similarly, at present, preoperative CTA radiomic feature stratification methods for EVAR were unavailable.

In this study, we first determined the significant morphological features and developed a morphological feature model based on different ML algorithms. We then used DL algorithms to make the model development more convenient. Subsequently, we studied the relationship between DL features, radiomic features, and EVAR-related severe adverse events (SAEs), to develop models and compare them with an optimized morphological feature model.

## Materials and Methods

### Data and Computed Tomography Collection

From January 2010 to December 2019, 1,523 patients with infrarenal AAAs who underwent elective EVAR at our single center were retrospectively reviewed. Patients with preoperative and postoperative CTA were enrolled in this study. The exclusion criteria were abdominal aortic dissecting aneurysm, ruptured AAA, isolated iliac artery aneurysm, history of aortic surgery, fenestration, and chimney technique. A total of 979 patients were included in this study. Ethical approval was obtained from the ethics committee of our hospital (B2021-063). The need to obtain informed consent from patients was waived due to the retrospective nature of the study.

Contrast-enhanced computed tomography (CT) was performed using Toshiba Aquilion One-64 (Version 3.1; Toshiba Medical Systems, Otawara, Japan) with 1-mm thickness slices. Triple-phase CT was performed, which included a plain scan, arterial phase, and portal venous phase. Arterial phase images were acquired 20–30 s after injection of contrast agent (Ultravist, Bayer Schering Pharma, Berlin, Germany).

### End Points

Patients were scheduled for follow-up at 3, 6, and 12 months and annually thereafter. CTA and color Doppler ultrasound were routinely performed. Follow-up data were collected until June 2021. The primary end points were EVAR-related SAEs, including type I/III endoleaks, persistent type II endoleaks (persist longer than 6 months), re-intervention, iliac limb occlusion or restenosis, stent-graft migration (≥5 mm), stent-graft fracture, aneurysm sac enlargement (≥5 mm), AAA rupture, and aneurysm-related mortality (3, 4).

### Morphological Feature Model Development

**FIGURE 1:**
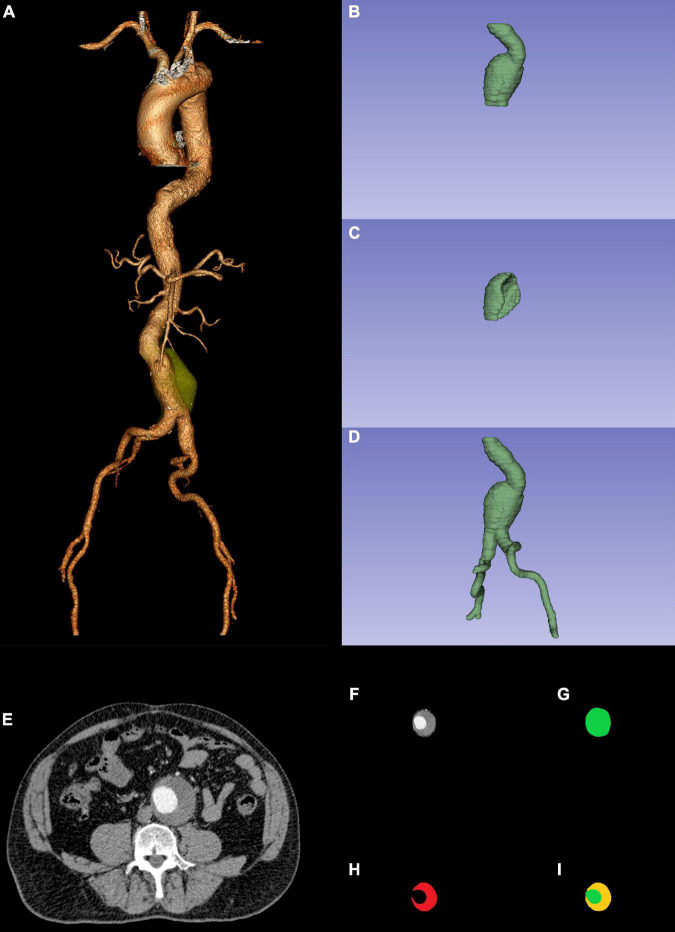
Preoperative CTA reconstruction **(A)**; AAA sac ROI **(B)**; ILT ROI **(C)**; AAA ROI **(D)**; AAA original ROI CTA slice **(F)** was the combination between AAA ROI CTA slicer **(G)** and original ROI CTA slice **(E)**; CTA slicer **(I)** was the combination between AAA sac ROI CTA slicer **(G)** and ILT ROI CTA slicer **(H)**. CTA, computed tomography angiography; AAA, abdominal aortic aneurysm; ROI, region of interest; ILT, intraluminal thrombosis.

Among the 979 patients, 486 patients (from January 2010 to December 2015) were used for morphological feature model development. Morphological features were extracted from the preoperative CTA images (Figure 1A). All of these morphological features were conducted with centerlines of flow by two investigators in a Vitrea Workstation, with disagreements resolved by a third one. All investigators were blinded to EVAR outcomes. A total of 32 morphological features were used in this study. The major morphological features included short neck (less than 15 mm), conical neck (neck dilation over 10% within 15 mm below the most caudal renal artery), angulated neck (at least 60^°^ between the long axis of the aneurysm sac and juxtarenal aorta), obvious thrombus (the widest part of thrombus (≥2 mm thick) covering at least 50% of the circumference of the proximal neck), calcified neck (calcification accounting for more than or equal to 50% of the proximal neck), ILT, ILT percentage, common iliac artery index (CAI), and double iliac sign (DIS) (26–28). The significant morphological features were used to build a morphological feature model based on different ML algorithms.

### Morphological Feature Model Optimization

To create a morphological feature model easier and compare it with other modal models, we used deep convolutional neural networks (DCNNs) to fully automatically segment ILT in preoperative CTA and realized automatic computation of ILT percentage (29, 30). To apply the DCNN model, AAA segmentation was performed in the preoperative CTA using ITK-SNAP (version 3.8.0^1^). The AAA sac region of interest (ROI: from the lowest renal artery to the aortic bifurcation) and ILT ROI were manually segmented by a junior vascular surgeon and adjusted by a senior vascular surgeon. We then combined the AAA sac ROI (Figures 1B,G) and the ILT ROI (Figures 1C,H). The combined ROI (Figure 1I) was resized to 512 × 512 pixels by third-order spline interpolation in each CTA slice and fed into the DCNN model. The software MATLAB (Version 2021a; MathWorks, Natick, Massachusetts) based on NVIDIA GeForce RTX 3090 was used to create the DCNN model.

We designed the relevant parameters and used a fully convolutional network named DeepLabv3+ semantic segmentation model (31) combined with a backbone convolutional feature extractor, the ResNet-50 network for transfer learning (32, 33), with the aim of automatic segmentation of the ILT and AAA aortic lumen (AL). A 4-fold cross-validation was employed to provide more robustness (34).

### Development and Comparison of Multimodal Models

The remaining 493 patients, from January 2016 to December 2019, were used for the development and comparison of multimodal models. To fully determine the nature of the training set and build a reliable classification model for further prediction, we used a 5-fold cross-validation technique (34).

#### An Optimized Morphological Feature Model

Based on previous statistical analysis results (significant morphological features) and the DCNN model (fully automatic segmentation ILT and realized automatic computation ILT percentage), in this section, we created a morphological feature model based on different ML algorithms, which became easier and more convenient.

#### Deep Learning Model Development

Segmentation of the AAA ROI (ROI: from the renal artery to the common femoral artery bifurcation) was performed in the same manner as the AAA sac ROI and ILT ROI. Since the AAA ROI (Figures 1D,G) represented less of the entire image, we normalized the non-ROI of the original images (Figure 1E) using MATLAB to obtain better predictions. Later, the AAA original ROI CTA slices (Figure 1F) were resized to 224 × 224 pixels by third-order spline interpolation in each CTA slice and fed into the DL model. The DL model development had the same environment as that of the DCNN model.

The ResNet-50 network was used for transfer learning (32, 33), and we only needed to modify the last number class to 2 and add the Sigmoid activation function after the fully connected layer. To make a robust prediction, each patient’s CTA slices of the AAA original ROI CTA slices were fed into the DL model, and the average probability was treated as the result of the probability of EVAR-related SAEs.

#### Radiomic Model Development

Radiomic features were extracted using Python (version 3.8.5^2^) through preoperative CTA images and the AAA ROI (Figure 1D). The Python packages used were SimpleITK (2.0.0), Sklearn (0.24.2), Pyradiomics (3.0.1), and PyWavelets (1.1.1). First, the Pearson correlation analysis excluded radiomic features with high reproducibility. We then used analysis of variance (ANOVA) to exclude features that had no significant influence on EVAR-related SAEs. Finally, the least absolute shrinkage and selection operator (LASSO) regression was used to determine the features related to SAEs after EVAR. The selected radiomic features were used to build models using different ML algorithms.

### Statistics Analysis

Descriptive analyses were performed to assess the clinical characteristics and outcomes of the cohort. Values are presented as frequencies or percentages for categorical features and as mean ± SD for continuous variables. Univariable and multivariable analyses were conducted to determine the significant morphological features of EVAR-related SAEs and to build a morphological feature model based on different ML algorithms. Based on the presence of EVAR-related SAEs, patients were randomly divided, 80% were used as the training set and 20% as the test set. The morphological feature model and radiomic model development were based on ML using Python. ML algorithms include logistics regression (LR), Naive Bayes (NB), k-nearest neighbors (KNN), decision tree (DT), SVM, random forest (RF), AdaBoost, Xgboost, and LightGBM. The DCNNs and DL models were developed using MATLAB software. The area under the curve (AUC) from the receiver-operating characteristic (ROC) curve was used to evaluate the predictive effect and select the best performing model in the training set. Applying the selected model, the AUC from the ROC curve was used to evaluate the predictive effect in the test set. Statistical significance was set at ***P*** < 0.05. Statistical analyses were performed using SPSS (Version 23, IBM, Armonk, NY, United States), Python, and MATLAB.

## Results

### Patient Characteristics and Outcomes

The mean age of the patients was 69.9 years (range: 41–89 years), and 779 of them were men (79.6%). Comorbidities included coronary heart disease (17.2%), hypertension (71.3%), hyperlipidemia (51.2%), and diabetes mellitus (8.9%). Of note, three types of modular devices were used in these patients, namely, 457 Endurant (Medtronic, Santa Rosa, CA, United States), 281 Excluder (W. L. Gore & Associates, Flagstaff, AZ, United States), and 241 Zenith (Cook Medical, Bloomington, IN, United States). The baseline demographic data were summarized in Table 1.

**TABLE 1:** Clinical characteristics and outcomes of patients with EVAR.

Variable	Patients (*n* = 979)
Age (years)	69.9 ± 8.1
Male sex *n* (%)	779 (79.6)
Coronary heart disease *n* (%)	168 (17.2)
Hypertension *n* (%)	698 (71.3)
Hyperlipidemia *n* (%)	501 (51.2)
Diabetes mellitus *n* (%)	87 (8.9)
Chronic obstructive pulmonary disease *n* (%)	275 (28.1)
Chronic renal failure *n* (%)	59 (12.1)
Peripheral artery disease *n* (%)	115 (11.7)
Endurant *n* (%)	457 (46.7)
Excluder *n* (%)	281 (28.7)
Zenith *n* (%)	241 (24.6)
Maximal aneurysm diameter (mm)	56.9 ± 15.3
Type Ia endoleak *n* (%)	35 (3.6)
Type Ib endoleak *n* (%)	37 (3.8)
Type III endoleak *n* (%)	7 (0.7)
Persistent type II endoleak *n* (%)	78 (8.0)
Iliac limb occlusion or restenosis *n* (%)	82 (8.4)
Sac enlargement *n* (%)	47 (4.8)
Aneurysm-related mortality *n* (%)	21 (2.1)

*EVAR, endovascular aneurysm repair.*

The mean follow-up was 54 months, including 307 (31.4%) patients who had EVAR-related SAEs, including 35 (3.6%) type Ia endoleak; 37 (3.8%) type Ib endoleak; 7 (0.7%) type III endoleak; 78 (8.0%) persistent type II endoleak; 82 (8.4%) iliac limb occlusion or restenosis; 47 (4.8%) sac enlargement; and 21 (2.1%) aneurysm-related mortality (Table 1).

### Morphological Feature Model Development and Test

**FIGURE 2:**
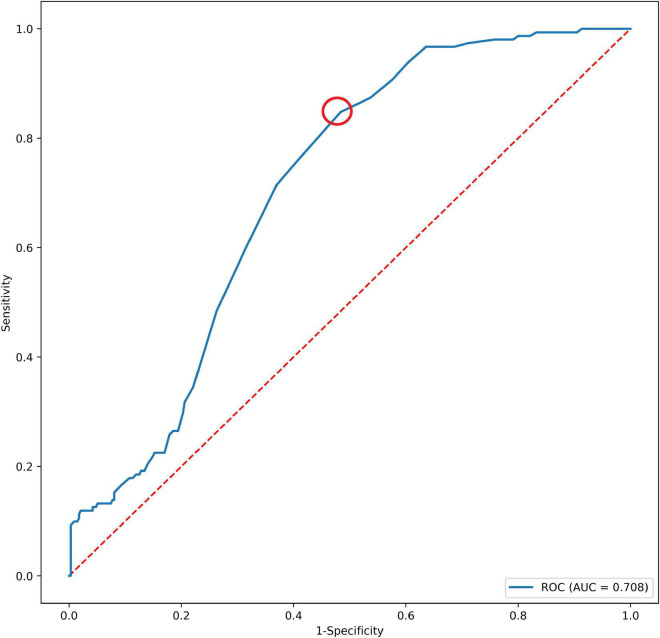
The AUC of CAI and SAEs after EVAR. The red dotted circle represents the optimal cutoff point. AUC, area under the curve; CAI, common iliac artery index; SAEs, severe adverse events.

In a total of 486 patients with a maximal Youden’s index of 0.364 (sensitivity: 0.848 and specificity: 0.484), the optimal cutoff value of the CAI was 1.255 (Figure 2). Multivariate analyses showed that short neck, angulated neck, conical neck, ILT, ILT percentage ≥51.6%, luminal calcification, DSI, and CAI (≥1.255) were significant morphological features of EVAR-related SAEs (Table 2).

**TABLE 2:** Predictors of SAEs after EVAR by multivariate analyses and predictive performance of different models.

Predictors of SAEs after EVAR by multivariate analyses				Predictive performance of different model in training set and test set					
Variable	OR	95% CI	*P*-value		AUC	Accuracy	Precision	Recall	F1 score
Age	1.005	0.978–1.033	0.714	LR Training set	0.79	0.76	0.90	0.79	0.84
Neck length	1.009	0.995–1.022	0.214	Test set	0.79	0.73	0.90	0.73	0.81
Short neck	0.598	0.364–0.983	0.042	KNN Training set	0.78	0.76	0.95	0.76	0.84
Angulated neck	1.079	1.020–1.142	0.009	Test set	0.75	0.69	0.98	0.67	0.80
Conical neck	2.440	1.465–4.064	0.001	DT Training set	0.78	0.74	0.92	0.77	0.84
ILT	0.425	0.206–0.874	0.020	Test set	0.72	0.68	0.90	0.68	0.77
ILT percentage ≥ 51.6%	8.024	2.715–23.71	0.000	SVM Training set	0.78	0.76	0.91	0.79	0.85
Luminal calcification	0.542	0.344–0.852	0.008	**Test set**	**0.76**	**0.76**	**0.90**	**0.75**	**0.82**
DSI	0.334	0.161–0.692	0.003	RF Training set	0.88	0.81	0.90	0.84	0.87
Common iliac calcification	0.573	0.296–1.109	0.099	Test set	0.66	0.66	0.83	0.68	0.75
CAI max	0.981	0.912–1.141	0.501	AdaBoost Training set	0.81	0.76	0.89	0.79	0.84
CAI (≥1.255)	2.404	1.394–4.148	0.002	Test set	0.79	0.73	0.88	0.74	0.80

*SAEs, severe adverse events; EVAR, endovascular aneurysm repair. The SVM had better predictive performance in the test set.*

A total of 388 patients were randomly assigned to the training set (No SAEs, 269; SAEs, 119); 98 patients were collected to the test set (No SAEs, 66; SAEs, 32). The AUC of the morphological feature model for predicting EVAR-related SAEs in the test set was as follows: LR 0.79, KNN 0.75, DT 0.72, SVM 0.76, RF 0.66, and AdaBoost 0.79. The accuracy for the test set was LR 0.73, KNN 0.69, DT 0.68, SVM 0.76, RF 0.66, and AdaBoost 0.73. The F1 scores in the test set were LR 0.81, KNN 0.80, DT 0.77, SVM 0.82, RF 0.75, and AdaBoost 0.80. The quantitative performance indicated that the morphological feature model based on SVM had a better predictive performance with an AUC of 0.76, an accuracy of 0.76, and an F1 score of 0.82 (Table 2).

### Morphological Feature Model Optimization

From January 2010 to December 2015, 340 patients of infrarenal AAAs with ILT were used for morphological feature model optimization. By training in 34,760–35,652 CTA images (*n* = 204) and validation in 6,968–7,860 CTA images (*n* = 68), our DCNN model achieved a mean intersection over union (IOU) more than 90.78% for ILT and AAA AL in test set (Table 3). The manual segmentation of ILT volume, AAA AL volume, and ILT percentage were 48.6 ± 9.779 cm^3^, 112.5 ± 77.63 cm^3^, and 30.18 ± 11.32%, respectively. Our DCNN model results were 46.8 ± 12.11 cm^3^, 107.1 ± 99.25 cm^3^, and 34.29 ± 10.70%. The ILT volume, AAA AL volume, and ILT percentage differences were less than 5% (3.81, 4.81, and 4.11%).

**TABLE 3:** The segmentation performance in the validation set and test set.

	Validation set	Test set
ILT IOU (mean ± SD)	0.8791 ± 0.0028	0.8650 ± 0.0033
AAA AL IOU (mean ± SD)	0.9108 ± 0.0050	0.8595 ± 0.0085
Mean IOU (mean ± SD)	0.9298 ± 0.0022	0.9078 ± 0.0029
ILT weight IOU (mean ± SD)	0.9981 ± 0.0001	0.9976 ± 0.0001

### Development and Comparison of Multimodal Models

A total of 493 patients were used for the development of multimodal models, 394 patients were allocated to the training set (No SAEs, 269; SAEs, 125), and 99 patients were allocated to the test set (No SAEs, 68; SAEs, 31). Multimodal models include optimized morphological features, DL, and radiomic models.

#### An Optimized Morphological Feature Model

**FIGURE 3:**
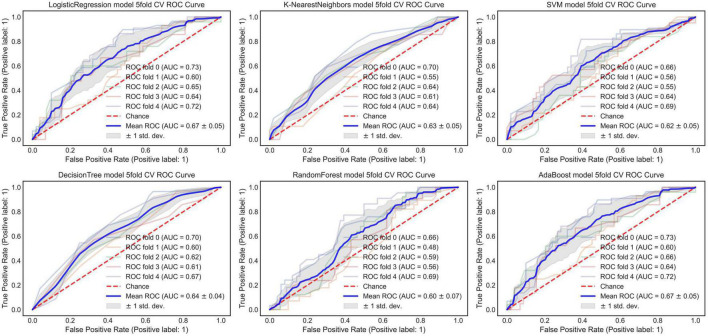
The AUC of the optimized morphological feature model for predicting EVAR-related SAEs in the training set. AUC, area under the curve; SAEs, severe adverse events; EVAR, endovascular aneurysm repair.

**FIGURE 4:**
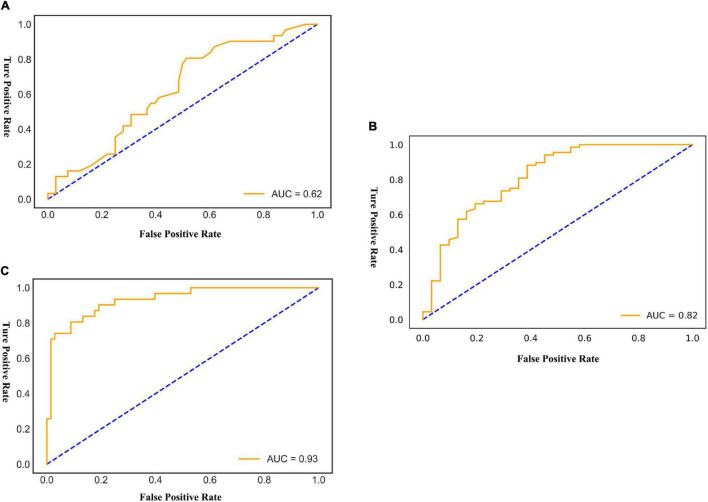
The AUC of the multimodal models for predicting EVAR-related SAEs in the test set. AUC, area under the curve; SAEs, severe adverse events; EVAR, endovascular aneurysm repair. **(A)** AdaBoost ROC curve with test dataset. **(B)** Fold 3 ROC curve with test dataset. **(C)** LogisticRegression ROC curve with test dataset.

The AUC of the optimized morphological feature model for predicting EVAR-related SAEs in the test set was as follows: LR 0.63, KNN 0.56, DT 0.56, SVM 0.58, RF 0.53, and AdaBoost 62. The accuracy for the test set was LR 0.60, KNN 0.71, DT 0.68, SVM 0.55, RF 0.56, and AdaBoost 0.69. The F1 score for the test set was LR 0.68, KNN 0.82, DT 0.80, SVM 0.61, RF 0.66, and AdaBoost 0.81. The ROC curves (Figures 3, 4A) and quantitative performance (Table 4) indicated that the optimized morphological feature model based on AdaBoost had a better predictive performance with an AUC of 0.62, an accuracy of 0.69, and an F1 score of 0.81.

**TABLE 4:** The predictive performance of multimodal models in the training set and test set.

		Training set		Test set				
		AUC	Accuracy	AUC	Accuracy	Precision	Recall	F1 score
Optimized morphological feature model	LR	0.67 ± 0.05 (0.60–0.73)	0.63 ± 0.05 (0.56–0.69)	0.63	0.60	0.75	0.62	0.68
	KNN	0.62 ± 0.04 (0.55–0.67)	0.67 ± 0.05 (0.59–0.73)	0.56	0.71	0.71	0.99	0.82
	DT	0.64 ± 0.04 (0.60–0.70)	0.68 ± 0.04 (0.62–0.72)	0.56	0.68	0.69	0.97	0.80
	SVM	0.62 ± 0.05 (0.55–0.69)	0.59 ± 0.05 (0.51–0.65)	0.58	0.55	0.74	0.51	0.61
	RF	0.59 ± 0.07 (0.48–0.70)	0.58 ± 0.05 (0.51–0.65)	0.53	0.56	0.70	0.62	0.66
	**AdaBoost**	**0.67 ± 0.05 (0.60–0.74)**	**0.68 ± 0.02 (0.65–0.71)**	**0.62**	**0.69**	**0.70**	**0.94**	**0.81**
Deep learning model	Fold 0	0.85	0.86	0.81	0.83	0.90	0.84	0.87
	Fold 1	0.89	0.89	0.81	0.84	0.89	0.87	0.88
	Fold 2	0.86	0.87	0.79	0.79	0.86	0.82	0.84
	**Fold 3**	**0.90**	**0.90**	**0.82**	**0.85**	**0.88**	**0.90**	**0.89**
	Fold 4	0.85	0.84	0.81	0.81	0.86	0.87	0.86
Radiomics model	**LR**	**0.93 ± 0.02 (0.90–0.95)**	**0.87 ± 0.03 (0.82–0.91)**	**0.93**	**0.86**	**0.94**	**0.89**	**0.91**
	NB	0.80 ± 0.03 (0.76–0.84)	0.77 ± 0.04 (0.73–0.83)	0.77	0.76	0.78	0.85	0.81
	SVM	0.93 ± 0.02 (0.89–0.95)	0.87 ± 0.04 (0.82–0.92)	0.92	0.86	0.93	0.88	0.90
	RF	0.93 ± 0.04 (0.87–0.97)	0.86 ± 0.04 (0.80–0.91)	0.90	0.89	0.96	0.89	0.92
	Xgboost	0.94 ± 0.03 (0.88–0.97)	0.87 ± 0.04 (0.81–0.92)	0.90	0.85	0.88	0.90	0.89
	LightGBM	0.94 ± 0.03 (0.89–0.97)	0.87 ± 0.04 (0.80–0.91)	0.90	0.86	0.88	0.91	0.89

*The AdaBoost, Fold 3 and LR had better predictive performance in the test set.*

#### Deep Learning Model

**FIGURE 5:**
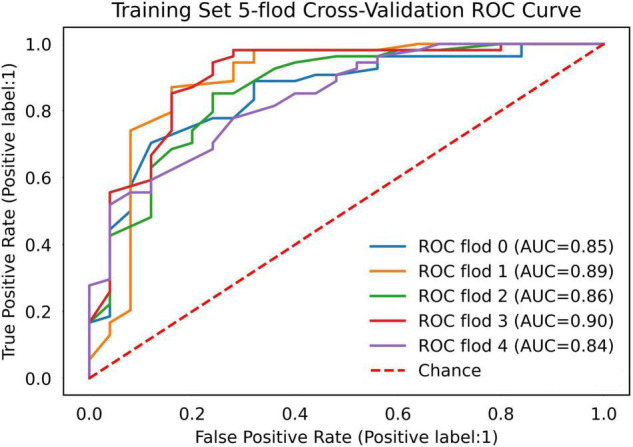
The AUC of the DL model for predicting EVAR-related SAEs in the training set. AUC, area under the curve; DL, deep learning; SAEs, severe adverse events; EVAR, endovascular aneurysm repair.

By training in 92012-93925 CTA images (*n* = 315), the DL model showed excellent predictive performance in validation set (*n* = 79) by 5-fold cross-validation with an AUC of 0.87 ± 0.03, accuracy of 0.87 ± 0.02, and F1 score of 0.90 ± 0.02. This performance was further confirmed in the test set (*n* = 79) with an AUC of 0.81 ± 0.01, an accuracy of 0.82 ± 0.02, and an F1 score of 0.87 ± 0.02. The ROC curves (Figures 4, 5) and quantitative performance (Table 4) indicated that the DL model (Fold 3) had a better predictive performance with an AUC of 0.82, an accuracy of 0.85, and an F1 score of 0.89.

#### Radiomic Model

**FIGURE 6:**
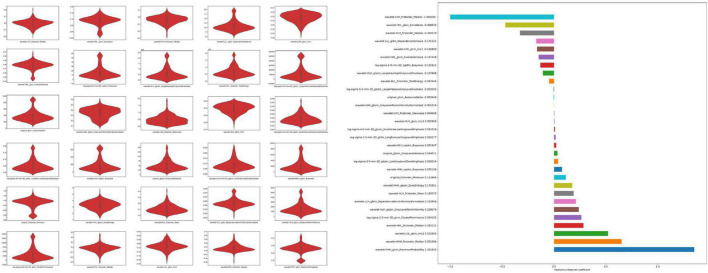
Significant radiomic features and feature coefficients.

**FIGURE 7:**
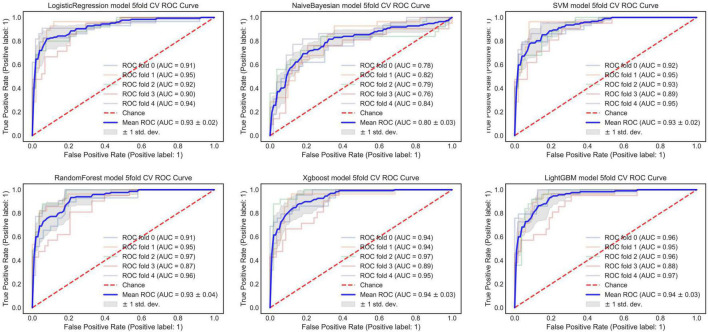
The AUC of the radiomic model for predicting EVAR-related SAEs in the training set. AUC, area under the curve; SAEs, severe adverse events; EVAR, endovascular aneurysm repair.

We extracted 1,223 radiomic features from each patient, and 30 features were preserved and used for radiomic model development. The significant radiomic features and feature coefficients are shown in Figure 6. The AUC of the radiomic model for predicting EVAR-related SAEs in the test set was as follows: LR 0.93, NB 0.77, SVM 0.92, RF 0.90, Xgboost 0.90, and LightGBM 0.90. The accuracy for the test set was LR 0.86, NB 0.76, SVM 0.86, RF 0.89, Xgboost 0.88, and LightGBM 0.88. The F1 score for the test set was LR 0.91, NB 0.81, SVM 0.90, RF 0.92, Xgboost 0.89, and LightGBM 0.89. The ROC curves (Figures 4, 7) and the quantitative performance (Table 4) indicated that the radiomic model based on LR had a better predictive performance with an AUC of 0.93, an accuracy of 0.86, and an F1 score of 0.91.

## Discussion

Existing risk score models, such as the Glasgow Aneurysm Score (GAS), Vascular Biochemical and Hematological Outcome Model (VBHOM), the National Surgical Quality Improvement Project (NSQIP), the Vascular Study Group of New England (VSGNE), the Vascular Governance North West model (VGNW), the Medicare model, and the EVAR Risk Assessment (ERA) model, cannot accurately predict complications and re-intervention in the era of EVAR (35–41). Fortunately, when studying the risk factors for model development, we found that morphological features were highly correlated with complications and re-intervention after EVAR. In addition, we also found that conventional statistical models could not fully exploit complex and subtle relationships between features for prediction. These findings were confirmed by Karthikesalingam Alan and Ali Kordzadeh. Karthikesalingam Alan applied ANN and 19 preoperative morphological features to predict endograft complications, and in external validation, the 5-year rates of freedom from aortic complications, limb complications, and mortality in low- and high-risk groups were 95.9 vs. 67.9%, 99.3 vs. 92.0%, and 87.9 vs. 79.3%, respectively (10). Ali Kordzadeh used ANN and 26 preoperative attributes (of which five morphological features) for the detection of endoleaks (types I–III, respectively), and the overall accuracy of the model was >86% (11). To avoid the limited number of patients with different complications that influenced the prediction performance and fully exploited morphological features, the prediction performance between different ML algorithms and model development was easier. A total of 486 patients were used for morphological feature model development, of which 151(31.1%) patients had SAEs. We identified 8 significant morphological features from 32 morphological features and developed a morphological feature model based on different ML algorithms. Our morphological feature model based on SVM had better performance than other ML algorithms, with an AUC of 0.76, an accuracy of 0.76, and an F1 score of 0.82. Later, we proposed the DCNN model for fully automatic segmentation of the ILT in preoperative CTA images. Distinct from traditional thrombus segmentation methods, which have been addressed with intensity-based semiautomatic algorithms combined with shape priors (42, 43). Our DCNN model fully automatically segmented ILT and achieved a mean IOU score of more than 90.78% for segmentation of ILT and AAA AL. The volume difference between the segmentation obtained with the proposed DCNNs and the ground truths was within the experienced human observer variance (*P* > 0.05). Both the ILT segmentation performance and ILT volume difference were better than the currently available models (44, 45).

Development and comparison of multimodal models showed that the optimized morphological feature model based on AdaBoost had a better predictive performance with an AUC of 0.62, an accuracy of 0.69, and an F1 score of 0.81. The AUC, accuracy, and F1 score decreased when compared with our previous studies (AUC 0.76, accuracy 0.76, and F1 score 0.82). We assumed that the main reason was that, in this section, we performed a 5-fold cross-validation, and the limited amount of data affected the model prediction performance (30). However, it did not prevent the morphological feature model as a useful adjunct tool for predicting outcomes after EAVR under the condition of sufficient data. In addition, the morphological feature model may be more acceptable for clinicians.

Based on DL algorithms and graphics processing units (GPUs) that power their training, the DL model has shown human expert-level performance in prognosis (12, 25). Our proposed DL model (Fold 3) predicts the outcome after EVAR with an AUC of 0.82, an accuracy of 0.85, and an F1 score of 0.89. Through a hierarchical neural network structure, the DL model extracted multilevel features from visual characteristics to abstract mappings that were directly related to SAEs after EVAR (29). The DL model was fast and easy to use. Since our method was an end-to-end pipeline that requires only the manually selected AAA region and image preprocessing, the CTA slice was input to predict the status after EVAR directly without further human input. It was easier than the morphological feature model and radiomic method. Besides, the DL model only required CTA images, without increasing the cost. In addition, CTA was easy to acquire throughout the course of treatment; therefore, this model could be used multiple times.

Radiomic methods used CT images to quantify AAA information at the macroscopic level, and radiomic analysis provides quantitative features to mine high-dimensional information and may build the relationship between AAA images and EVAR-related SAEs (20–22). Reviewing the current literature, few radiomic studies have been carried out in patients with AAA. However, there have been many studies about texture analysis, and Carl W. Kotze used CT texture analysis and 18F-fluorodeoxyglucose positron emission tomography to predict AAA dilatation (16). García G developed a computer-supported endotension detection method based on texture analysis for EVAR (17). García G also evaluated the texture features and classification of AAA endoleak after EVAR, gray-level co-occurrence matrix (GLCM), gray-level run-length matrix (GLRLM), and gray-level dependence matrix (GLDM) were able to distinguish favorable or unfavorable regression with an accuracy of 93.41 ± 0.024%, 90.17 ± 0.077%, and 81.98 ± 0.045%, respectively (18). Ding N applied GLCM, GLRLM, and GLDM for predicting aneurysm expansion after EVAR with accuracy (85.17, 87.23, and 86.09%) and AUC (0.90, 0.86, and 0.83) (19). Recently, Charalambous Stavros used radiomics and the SVM classifier trained on 6-month radiomic features to predict T2ELs leading sac expansion at 1 year with an AUC of 89.3%, and the SVM classifier developed with 6-month radiomic features showed an AUC of 95.5% at 1 year (23). Our radiomic model is based on LR to preoperative prediction outcomes after EVAR with AUC 0.93, accuracy 0.86, and F1 score 0.91. Although, compared with other modal models, the radiomic model had better prediction performance. We suggest that the morphological feature, DL, and radiomic models all can be used to predict outcomes after EVAR.

Our study has some limitations. First, due to the retrospective single-center nature of the study, selective bias could not be avoided. Despite internal validation and testing, it was necessary to estimate these multimodal models at other institutions. Second, the limited data affected the prediction performance for the development of multimodal models. Besides, the relatively small sample size limited the possibility of conducting a subgroup predicting different SAEs after EVAR. Third, the morphological feature model development was still time-consuming under the condition of fully automatically segmenting ILT and computing ILT percentage. The DL model showed effective performance for predicting outcomes after EVAR. A high environment was required for DL model development. At present, radiomics lacks a standardized methodology for radiomic analyses, a universal lexicon to denote features that are semantically equivalent, and lists of feature values alone do not sufficiently capture the details of feature extraction that might nonetheless strongly affect feature values (43, 44). Finally, the combination of morphological features, DL, and radiomic features was unclear. In the future study, we will aim to improve prediction performance by combining different modal models.

## Conclusion

Applying morphological features, DL, and radiomic models, we can evaluate the risk of postoperative outcomes after EVAR. Using these models, operators can more accurately estimate individual patient risk after EVAR and identify subgroups of patients who require more intensive follow-up. This might affect both patient selection and surveillance after EVAR, which remained important for prognosis after EVAR.

## Data Availability Statement

The original contributions presented in the study are included in the article/Supplementary Material, further inquiries can be directed to the corresponding author/s.

## Ethics Statement

The studies involving human participants were reviewed and approved by Zhongshan Hospital Fudan University Ethic Committee. Written informed consent for participation was not required for this study in accordance with the national legislation and the institutional requirements.

## Author Contributions

ZS and WF contributed to the conception and design. MZ, YD, and XL performed the data collection and interpretation. YW, ZZ, and ZS performed ROI segmentation and images preprocessing. YW analyzed the datasets and wrote this manuscript. ZS obtained the funding. All authors read and approved the final manuscript.

## Conflict of Interest

The authors declare that the research was conducted in the absence of any commercial or financial relationships that could be construed as a potential conflict of interest. The handling editor ZL is currently organizing a Research Topic with WF.

## Publisher’s Note

All claims expressed in this article are solely those of the authors and do not necessarily represent those of their affiliated organizations, or those of the publisher, the editors and the reviewers. Any product that may be evaluated in this article, or claim that may be made by its manufacturer, is not guaranteed or endorsed by the publisher.

## Footnotes

1
http://www.itksnap.org

2
http://www.python.org

## References

[1] Patel R Sweeting MJ Powell JT Greenhalgh RM. Endovascular versus open repair of abdominal aortic aneurysm in 15-years’ follow-up of the UK endovascular aneurysm repair trial 1 (EVAR trial 1): a randomised controlled trial. *Lancet.* (2016) 388:2366–74. 10.1016/S0140-6736(16)31135-7 27743617

[2] Lederle FA Kyriakides TC Stroupe KT Freischlag JA Padberg FT Jr. Matsumura JS Open versus endovascular repair of abdominal aortic aneurysm. *N Engl J Med.* (2019) 380:2126–35. 10.1056/NEJMoa1715955 31141634

[3] Chaikof EL Dalman RL Eskandari MK Jackson BM Lee WA Mansour MA The society for vascular surgery practice guidelines on the care of patients with an abdominal aortic aneurysm. *J Vasc Surg.* (2018) 67:2–77. 10.1016/j.jvs.2017.10.044 29268916

[4] Wanhainen A Verzini F Van Herzeele I Allaire E Bown M Cohnert T Editor’s choice – European society for vascular surgery (ESVS) 2019 clinical practice guidelines on the management of abdominal aorto-iliac artery aneurysms. *Eur J Vasc Endovasc Surg.* (2019) 57:8–93. 10.1016/j.ejvs.2018.09.020 30528142

[5] Nana P Kouvelos G Brotis A Spanos K Giannoukas A Matsagkas M. The effect of endovascular aneurysm repair on renal function in patients treated for abdominal aortic aneurysm. *Curr Pharm Des.* (2019) 25:4675–85. 10.2174/1381612825666191129094923 31782360

[6] Harbron RW Abdelhalim M Ainsbury EA Eakins JS Alam A Lee C Patient radiation dose from x-ray guided endovascular aneurysm repair: a Monte Carlo approach using voxel phantoms and detailed exposure information. *J Radiol Prot.* (2020) 40:704–26. 10.1088/1361-6498/ab944e 32428884

[7] Burgers LT Vahl AC Severens JL Wiersema AM Cuypers PW Verhagen HJ Cost-effectiveness of elective endovascular aneurysm repair versus open surgical repair of abdominal aortic aneurysms. *Eur J Vasc Endovasc Surg.* (2016) 52:29–40. 10.1016/j.ejvs.2016.03.001 27118618

[8] Steuer J Lachat M Veith FJ Wanhainen A. Endovascular grafts for abdominal aortic aneurysm. *Eur Heart J.* (2016) 37:145–51. 10.1093/eurheartj/ehv593 26543044

[9] Oliveira NFG Gonçalves FB Hoeks SE Josee van Rijn M Ultee K Pinto JP Long-term outcomes of standard endovascular aneurysm repair in patients with severe neck angulation. *J Vasc Surg.* (2018) 68:1725–35. 10.1016/j.jvs.2018.03.427 29914837

[10] Fujii T Banno H Kodama A Sugimoto M Akita N Tsuruoka T Aneurysm sac thrombus volume predicts aneurysm expansion with type II endoleak after endovascular aneurysm repair. *Ann Vasc Surg.* (2020) 66:85–94. 10.1016/j.avsg.2019.11.045 31863957

[11] Mascoli C Faggioli G Gallitto E Longhi M Abualhin M Pini R Planning and endograft related variables predisposing to late distal type I endoleaks. *Eur J Vasc Endovasc Surg.* (2019) 58:334–42. 10.1016/j.ejvs.2019.03.036 31358363

[12] Karthikesalingam A Attallah O Ma X Bahia SS Thompson L Vidal-Diez A An artificial neural network stratifies the risks of re-intervention and mortality after endovascular aneurysm repair; a retrospective observational study. *PLoS One.* (2015) 10:e0129024. 10.1371/journal.pone.0129024 26176943 PMC4503678

[13] Kordzadeh A Hanif MA Ramirez MJ Railton N Prionidis I Browne T. Prediction, pattern recognition and modelling of complications post-endovascular infra renal aneurysm repair by artificial intelligence. *Vascular.* (2021) 29:171–82. 10.1177/1708538120949658 32829694

[14] Tran KA Kondrashova O Bradley A Williams ED Pearson JV Waddell N. Deep learning in cancer diagnosis, prognosis and treatment selection. *Genome Med.* (2021) 13:152. 10.1186/s13073-021-00968-x 34579788 PMC8477474

[15] Gao R Zhao S Aishanjiang K Cai H Wei T Zhang Y Deep learning for differential diagnosis of malignant hepatic tumors based on multi-phase contrast-enhanced CT and clinical data. *J Hematol Oncol.* (2021) 14:154. 10.1186/s13045-021-01167-2 34565412 PMC8474892

[16] Esteva A Kuprel B Novoa RA Ko J Swetter SM Blau HM Dermatologist-level classification of skin cancer with deep neural networks. *Nature.* (2017) 542:115–8. 10.1038/nature21056 28117445 PMC8382232

[17] Bera K Braman N Gupta A Velcheti V Madabhushi A. Predicting cancer outcomes with radiomics and artificial intelligence in radiology. *Nat Rev Clin Oncol.* (2022) 19:132–46. 10.1038/s41571-021-00560-7 34663898 PMC9034765

[18] Kotze CW Rudd JHF Ganeshan B Menezes LJ Brookes J Agu O CT signal heterogeneity of abdominal aortic aneurysm as a possible predictive biomarker for expansion. *Atherosclerosis.* (2014) 233:510–7. 10.1016/j.atherosclerosis.2014.01.001 24530787

[19] García G Tapia A De Blas M. Computer-supported diagnosis for endotension cases in endovascular aortic aneurysm repair evolution. *Comput Methods Programs Biomed.* (2014) 115:11–9. 10.1016/j.cmpb.2014.03.004 24721658

[20] García G Maiora J Tapia A De Blas M. Evaluation of texture for classification of abdominal aortic aneurysm after endovascular repair. *J Digit Imaging.* (2012) 25:369–76. 10.1007/s10278-011-9417-7 21901536 PMC3348989

[21] Ding N Hao Y Wang Z Xuan X Kong L Xue H CT texture analysis predicts abdominal aortic aneurysm post-endovascular aortic aneurysm repair progression. *Sci Rep.* (2020) 10:12268. 10.1038/s41598-020-69226-1 32703988 PMC7378225

[22] Lambin P Rios-Velazquez E Leijenaar R Carvalho S van Stiphout RG Granton P Radiomics: extracting more information from medical images using advanced feature analysis. *Eur J Cancer.* (2012) 48:441–6. 10.1016/j.ejca.2011.11.036 22257792 PMC4533986

[23] Kumar V Gu Y Basu S Berglund A Eschrich SA Schabath MB Radiomics: the process and the challenges. *Magn Reson Imaging.* (2012) 30:1234–48. 10.1016/j.mri.2012.06.010 22898692 PMC3563280

[24] Gillies RJ Kinahan PE Hricak H. Radiomics: images are more than pictures. They are data. *Radiology.* (2016) 278:563–77. 10.1148/radiol.2015151169 26579733 PMC4734157

[25] Charalambous S Klontzas ME Kontopodis N Ioannou CV Perisinakis K Maris TG Radiomics and machine learning to predict aggressive type 2 endoleaks after endovascular aneurysm repair: a proof of concept. *Acta Radiol.* (2021) 27:2841851211032443. 10.1177/02841851211032443 34313492

[26] Brownrigg JR de Bruin JL Rossi L Karthikesalingam A Patterson B Holt PJ Endovascular aneurysm sealing for infrarenal abdominal aortic aneurysms: 30-day outcomes of 105 patients in a single centre. *Eur J Vasc Endovasc Surg.* (2015) 50:157–64. 10.1016/j.ejvs.2015.03.024 25892319

[27] Ding Y Shan Y Zhou M Cai L Li X Shi Z Amount of intraluminal thrombus correlates with severe adverse events in abdominal aortic aneurysms after endovascular aneurysm repair. *Ann Vasc Surg.* (2020) 67:254–64. 10.1016/j.avsg.2020.02.011 32173473

[28] Taudorf M Jensen LP Vogt KC Grønvall J Schroeder TV Lönn L. Endograft limb occlusion in EVAR: iliac tortuosity quantified by three different indices on the basis of preoperative CTA. *Eur J Vasc Endovasc Surg.* (2014) 48:527–33. 10.1016/j.ejvs.2014.04.018 24878235

[29] LeCun Y Bengio Y Hinton G. Deep learning. *Nature.* (2015) 521:436–44. 10.1038/nature14539 26017442

[30] Shelhamer E Long J Darrell T. Fully convolutional networks for semantic segmentation. *IEEE Trans Pattern Anal Mach Intell.* (2017) 39:640–51. 10.1109/TPAMI.2016.2572683 27244717

[31] Chen L-C Zhu Y Papandreou G Schroff F Adam H. Encoder-decoder with atrous separable convolution for semantic image segmentation. In: Ferrari V Hebert M Sminchisescu C Weiss Y editors. *Computer Vision – ECCV 2018. Lecture Notes in Computer Science.* Cham: Springer (2018).

[32] Raghu S Sriraam N Temel Y Rao SV Kubben PL. EEG based multi-class seizure type classification using convolutional neural network and transfer learning. *Neural Netw.* (2020) 124:202–12. 10.1016/j.neunet.2020.01.017 32018158

[33] Joshi V Le Gallo M Haefeli S Boybat I Nandakumar SR Piveteau C Accurate deep neural network inference using computational phase-change memory. *Nat Commun.* (2020) 11:2473. 10.1038/s41467-020-16108-9 32424184 PMC7235046

[34] Qin LX Huang HC Begg CB. Cautionary note on using cross-validation for molecular classification. *J Clin Oncol.* (2016) 34:3931–8. 10.1200/JCO.2016.68.1031 27601553 PMC5477984

[35] Patterson BO Karthikesalingam A Hinchliffe RJ Loftus IM Thompson MM Holt PJ. The glasgow aneurysm score does not predict mortality after open abdominal aortic aneurysm in the era of endovascular aneurysm repair. *J Vasc Surg.* (2011) 54:353–7. 10.1016/j.jvs.2011.01.029 21458200

[36] Patterson BO Holt PJ Hinchliffe R Nordon IM Loftus IM Thompson MM. Existing risk prediction methods for elective abdominal aortic aneurysm repair do not predict short-term outcome following endovascular repair. *J Vasc Surg.* (2010) 52:25–30. 10.1016/j.jvs.2010.01.084 20434296

[37] Eslami MH Rybin DV Doros G Farber A. Description of a risk predictive model of 30-day postoperative mortality after elective abdominal aortic aneurysm repair. *J Vasc Surg.* (2017) 65:65–74. 10.1016/j.jvs.2016.07.103 27720320

[38] Eslami MH Rybin D Doros G Kalish JA Farber A. Comparison of a Vascular Study Group of New England risk prediction model with established risk prediction models of in-hospital mortality after elective abdominal aortic aneurysm repair. *J Vasc Surg.* (2015) 62:1125–33. 10.1016/j.jvs.2015.06.051 26187291

[39] Grant SW Grayson AD Purkayastha D Wilson SD McCollum C. Logistic risk model for mortality following elective abdominal aortic aneurysm repair. *Br J Surg.* (2011) 98:652–8. 10.1002/bjs.7463 21412997

[40] Giles KA Schermerhorn ML O’Malley AJ Cotterill P Jhaveri A Pomposelli FB Risk prediction for perioperative mortality of endovascular vs open repair of abdominal aortic aneurysms using the Medicare population. *J Vasc Surg.* (2009) 50:256–62. 10.1016/j.jvs.2009.01.044 19249184 PMC2785461

[41] Barnes M Boult M Maddern G Fitridge R. A model to predict outcomes for endovascular aneurysm repair using preoperative variables. *Eur J Vasc Endovasc Surg.* (2008) 35:571–9. 10.1016/j.ejvs.2007.12.003 18255324

[42] Lalys F Yan V Kaladji A Lucas A Esneault S. Generic thrombus segmentation from pre- and post-operative CTA. *Int J Comput Assist Radiol Surg.* (2017) 12:1501–10. 10.1007/s11548-017-1591-8 28455765

[43] Zohios C Kossioris G Papaharilaou Y. Geometrical methods for level set based abdominal aortic aneurysm thrombus and outer wall 2D image segmentation. *Comput Methods Programs Biomed.* (2012) 107:202–17. 10.1016/j.cmpb.2011.06.009 21880391

[44] López-Linares K Aranjuelo N Kabongo L Maclair G Lete N Ceresa M Fully automatic detection and segmentation of abdominal aortic thrombus in post-operative CTA images using deep convolutional neural networks. *Med Image Anal.* (2018) 46:202–14. 10.1016/j.media.2018.03.010 29609054

[45] Caradu C Spampinato B Vrancianu AM Bérard X Ducasse E. Fully automatic volume segmentation of infrarenal abdominal aortic aneurysm computed tomography images with deep learning approaches versus physician controlled manual segmentation. *J Vasc Surg.* (2021) 74:246.e–56.e6. 10.1016/j.jvs.2020.11.036 33309556

[46] Shi Z Traverso A van Soest J Dekker A Wee L. Technical Note: ontology-guided radiomics analysis workflow (O-RAW). *Med Phys.* (2019) 46:5677–84. 10.1002/mp.13844 qrPlease cite Refs. (46, 47) inside the text. 31580484 PMC6916323

[47] van Timmeren JE Cester D Tanadini-Lang S Alkadhi H Baessler B. Radiomics in medical imaging-“how-to” guide and critical reflection. *Insights Imaging.* (2020) 11:91. 10.1186/s13244-020-00887-2 32785796 PMC7423816

